# Time management disposition and relevant factors among new nurses in Chinese tertiary hospitals: A cross-sectional study

**DOI:** 10.3389/fpsyg.2022.956945

**Published:** 2022-08-12

**Authors:** Jianfei Xie, Xiaoqi Wu, Jie Li, Xiaolian Li, Panpan Xiao, Sha Wang, Zhuqing Zhong, Siqing Ding, Jin Yan, Lijun Li, Andy S. K. Cheng

**Affiliations:** ^1^Nursing Department, Third Xiangya Hospital, Central South University, Changsha, China; ^2^Xiangya School of Nursing, Central South University, Changsha, China; ^3^The Hong Kong Polytechnic University, Hong Kong, Hong Kong SAR, China

**Keywords:** new nurses, time management disposition, job stressors, clinical communication, self-efficacy

## Abstract

**Introduction:**

New nurses struggled with time management, which was a prominent theme in safety care for patients. However, the transition training of time management for new nurses was complicated and ignored by clinical managers. The purpose of this study was to understand the level of new nurses’ TMD from a nationwide perspective and detect the influencing factors of the TMD.

**Materials and methods:**

A cross-sectional study design with a stratified sampling method was sampled in China. Six hundred and seventy new nurses within the first year of employment were recruited. New nurses’ time management disposition, job stressors, self-efficacy, clinical communication competence, and safety behavior were measured by corresponding scales.

**Results:**

New nurses showed the best sense of time’s value, followed by the sense of time efficacy and time monitoring view for time management disposition. The related factors of time management disposition were communication skills, safety behavior, job stressors, and being without a preceptor. New nurses’ time management disposition was at a moderate level and they performed worse in time allocation. The highest education, with or without a preceptor, the experience of part-time jobs, and class cadre were significantly influencing the time management disposition of new nurses.

**Conclusion:**

Nursing managers should pay attention to new nurses’ time management disposition. Reducing the job stressors, improving communication ability, and safe behavior were important measures to improve the time management disposition.

## Introduction

New nurses refer to the group of nurses who have just graduated from school and entered clinical work within one year. When they first enter the clinical environment, many new nurses experienced a theory-practice gap (Monaghan, 2015). Effective transition programs were an important support system for new nurses to get through the transition period, which is also complicated for clinical managers. New nurses face the transition stressors from nursing theories to clinical practice, especially during the first year (Della Ratta, 2016). They must strive to prioritize patient care, identify and control patient problems, understand the rationale for approaches to these problems, and engage in meaningful communication with medical colleagues and patients. Owing to the heavy workload, they encounter low to moderate levels of work stress (Wallace and Boller, 2014; Labrague and McEnroe-Petitte, 2018; Powers et al., 2019). For new nurses, work stress can also increase time management despair (Murray et al., 2019). These may be the reason why new nurses were prone to adverse events during their first practice year (Africa and Shinners, 2020).

In various execution and management fields, time management is a broad concept that is related to improving the quality performance of managers. Enable individuals or organizations to use time more effectively and improve time utilization to achieve their goals (Macan et al., 2010). Rational time management is positively associated with job performance, provides more time to complete higher priority tasks, and accelerates the progress of activities, enhancing individuals’ job satisfaction (Ahmad et al., 2012; Kaya et al., 2012; Bjerregård Madsen et al., 2016). Meanwhile, effective time management can reduce work stress, influence perceived effectiveness to some extent (Grissom et al., 2015).

Time management ability is one of the eight conceptual competencies of new nurses (Ramritu and Barnard, 2001). Many new nurses feel pressed for time and struggle with time management, which was a kind of key stressor during work (Knowlton, 2017; Murray et al., 2019). Improving time management ability contributed to the priorities of patients’ care in clinical nursing (Algoso et al., 2019). The stress of medication administration is the main problem for new nurses’ time management ability (Murray et al., 2019, 2020), which may result in completion tasks usually being ranked higher priority than patients’ safety. Time management was a prominent theme in providing safe care to patients for new nurses (Huang and Zhang, 2001) proposed the concept of the psychological and behavioral characteristics of individuals in the time management–time management disposition (TMD), which is a personality trait with a multi-dimensional and multi-level psychological structure. This personality trait is dynamic and has different degrees of performance, cross-situation, and potential measurability in different populations.

The above research mainly investigates the time management ability of new nurses in clinical situations, emphasized performance, stressors, and clinical safety (Murray et al., 2019, 2020). New nurses’ psychological feature of time management was rarely researched. A previous study demonstrated a strong relationship between time management experience and conscientiousness and academic achievement (MacCann et al., 2012). Furthermore, the time management disposition is predicted by general self-efficacy and self-control character among nursing managers (Xie et al., 2020). We discovered that the Nurses Job Stressors Scale (NJSS) with a dimension of time allocation and workload has a great impact on the overall stress. When we designed the questionnaire, we chose a special TMD scale to explore time management problems (Xiamei and Yanjun, 2000; Huang and Zhang, 2001). Communication skills and patient safety are important job stressors for new nurses (Kowalski and Cross, 2010). Clinical communication and safety perception are also connected with personality traits (Sathe and Sathe, 2015; Zaninotto et al., 2018). We attempt to explore the connection between corresponding job stressors and TMD among new nurses.

The connection between job stressors, communication ability, safety behavior, and TMD in new nurses was still unclear. The current published literature, such as those works discussed above, concentrated on nursing students, nurse managers, or general nurses. Managerial experience, depression, work stress, job satisfaction, and work competence have shown a strong correlation to nurses’ TMD (Jvsu, 2011; Liu et al., 2014; Xie et al., 2020). These factors that influence new nurses’ TMD have not been extensively examined. Therefore, the purposes of this study were to (1) understand the level of new nurses’ TMD from a nationwide perspective and (2) detect how the relevant factors influence new nurses’ TMD.

## Materials and methods

### Design

The design was a cross-sectional study. A stratified sampling method was used to obtain the final samples.

### Participants and procedure

We obtained detailed information on the number and proportion of registered nurses (RNs) in each region of China from the 2012 China Health Statistics released by the National Health and Family Planning Commission, and obtained details of hospital-level in each region of China from the following websites: National Health and Family Planning Commission of the People’s Republic of China.^1^ In addition, public hospitals are categorized into three levels (1st, 2nd, and 3rd) according to the availability of advanced medical equipment, the number of inpatient beds, the physical size of the medical campus, etc. (Xie et al., 2017). This study mainly focused on the tertiary hospitals, which are comprehensive or general hospitals at the city, provincial or national level with a bed capacity exceeding 500. They are larger and well equipped than 1st and 2nd level hospitals. We selected 20 3rd-level hospitals to participate in the study, these hospitals were located in 15 cities within 13 provinces from seven geographical areas.

**FIGURE 1:**
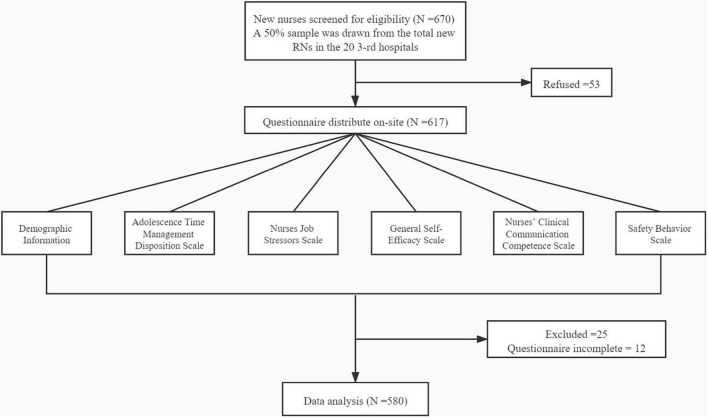
Work flowchart of new nurses through this study.

In China, new nurses are generally employed in the hospitals from June to July every year. We chose to survey between August and September 2019, which is the period of new nurses who have worked for 2–3 months. Firstly, the project leader contacted the heads of nursing departments of each hospital through the communication platform of the China Nursing Association and invited each hospital to participate. Then the nursing directors randomly selected 50% of the new nurses according to their professional level. Finally, we gathered all the potential participants, explained the purpose of the study, and obtained their consent. The inclusion criteria were: (1) newly employed nurses in 2019; (2) who provided direct care to patients; and (3) who were Chinese speakers. The investigation was performed among direct care nurses from clinical units, excluding drug distribution centers, disinfection supply centers, etc. Moreover, participating nurses should finish informed consent before completing the questionnaire (Figure 1).

### Instruments

#### Demographic information

A 12-item demographic questionnaire was applied to collect the gender, department, age, birthplace, highest education, marital status, and experience of work, etc.

#### Adolescence Time Management Disposition Scale

The TMD was evaluated by the ATMD, which was developed by Huang and Zhang (2001) in China and contained 44 items scored on a 5-point Likert scale (1 = not exactly at all, 5 = exactly). It encompasses three subscales: the sense of time’s value with 10 items (individual orientation, social orientation), time monitoring view with 24 items (offered target, plans, priorities, feedback, and time allocations), and the sense of time efficacy with 10 items (time management efficacy and time management behavior efficacy). Sense of time’s value refers to the individual’s stable attitude and viewpoint on the function and value of time, which plays a guiding role in the way individuals use time and is the basis of individual time management. The time monitoring view is the concept and ability of individuals to utilize and operate time, which is reflected through a series of explicit activities. Sense of time efficacy is the belief and expectation of individuals in their use and operation of time, demonstrates the individual’s confidence in time management and the estimation of time management behavior ability, it is an important factor restricting time monitoring (Huang and Zhang, 2001). The total score is calculated from 44 to 220 points. A higher score indicated a greater level of TMD. The average scores of ATMD between 1.00 and 2.33 indicated a low level, 2.34–3.66 means a medium level, and 3.67–5.00 means a high level (Jvsu, 2011). The scale has good reliability and validity, the internal consistency coefficient is 0.610–0.810, and the retest reliability is 0.710–0.850, which is widely used in the Chinese new group of nursing.

#### Nurses Job Stressors Scale

Li and Liu (2000) designed the Chinese version based on the two most common nurse job stressors scales by the American and British experts (Dolan, 1987; Wheeler and Riding, 1994; Xiamei and Yanjun, 2000). It consists of 35 items within 5 subscales nursing professional and work issues (7 items), time allocation and workload issues (5 items), work environment and equipment problems (3 items), patient care problems (11 items), management and interpersonal problems (9 items). The NJSS are rated on a 4-point Likert. Higher scores indicate more stress. The Cronbach’s α coefficient of 5 subscales was 0.830–0.980 (Xiamei and Yanjun, 2000).

#### General Self-Efficacy Scale

The General Self-Efficacy Scale (GSES) was originally developed by Schwarzer et al. (1992) and reflected the optimistic self-belief. Zhang and Schwarzer (1995) developed the Chinese version of the GSES to evaluate general self-efficacy among the general population. This version contains 10 items, with a score range of 1–4 points for each item. Higher scores represent higher levels of efficacy. It has been shown to have a Cronbach’s α coefficient of 0.870, split-half reliability of 0.820, and retest reliability of 0.830 (Wang et al., 2001).

#### Nurses’ Clinical Communication Competence Scale

The Nurses’ Clinical Communication Competence Scale (NCCCS) was first developed to measure the communication ability of clinical nurses in China (Kai, 2010). This was a 58-items scale, scored on a 5-Likert (1 = very poor, 5 = very good). The lower the score, the worse the clinical communication ability of the nurses. It has been shown to have a Cronbach’s α value on the scale was 0.978, and the retest reliability was 0.727 (Kai, 2010).

#### Safety Behavior Scale

The Chinese version of Safety Behavior Scale (SBS) was revised based on the safety behavior questionnaire prepared by Shih et al. (2008) and Yanfu and Zhengyi (2009). In this study, we applied the 9-item scale updated by Yang et al. (2017), Shuhong et al. (2017). The SBS can understand the behaviors of nurses in their work to avoid harm to patients or improve patient safety. It was scored using the Likert 5-point scoring method. “Never” scored 1 and “Always” scored 5. The higher the score, the better the nurse’s performance in patient safety. The Cronbach’s α coefficient of the 9-item SBS in nurses was 0.805 and was widely applied in China (Shuhong et al., 2017).

### Data analysis

The data were analyzed by the SPSS Statistics 23.0 program for Windows (IBM Corp., Armonk, NY, United States). Frequency and percentage, means and standard deviations were used to describe the classification and continuous variables. Because the data were non-normal, to identify the independent variables that correlated with the TMD, Kruskal–Wallis *H* test, Wilcoxon rank-sum test, and Spearman’s rank correlation analysis were used. The categorical data were transformed into dummy variables because the results of ATMD were continuous variables. Stepwise regression analysis was applied to investigate the predictions of the new nurses’ TMD. A value of *p* < 0.05 was considered statistically significant.

## Results

### Demographic characteristics and their correlation to time management disposition

A total of 670 questionnaires were distributed to eligible participants, and 580 (86.57%) valid questionnaires were obtained. As shown in Table 1, of the 580 new nurses, 94.7% were female, the average age was 22.15 ± 0.71 years old, 86.0% were unmarried, and 82.4% were native province people. Among them, 46.2% were born in rural areas. With regard to education, 64.6% have a bachelor’s degree or higher. In terms of past work-study experience, only 36.9% of them have a preceptor when they first enter the clinical environment, 18.6% have been a class cadre, and 20.9% have worked part-time jobs. For the relationship between the total score of ATMDS and the new nurses’ characteristics, there was a significant difference between ATMD and highest education (χ^2^ = 15.056, *p* = 0.002), with or without a preceptor, the experience of the class cadre, and part-time jobs (*Z* = –4.000, –2.039, and –2.584, respectively, *p* < 0.05).

**TABLE 1:** Demographic characteristics and differences in time management disposition (*N* = 580).

Characteristics		*N* (%)/ Mean ± SD	ATMD	Z/χ^2^	*p*
			Mean ± SD		
Gender	Male	31 (5.3)	3.63 ± 0.31	–1.657	0.098
	Female	549 (94.7)	3.53 ± 0.42		
Age, y		22.15 ± 0.71			
Birthplace	Urban	145 (25.0)	3.55 ± 0.41	4.464	0.216
	County	53 (9.1)	3.48 ± 0.45		
	Town	114 (19.1)	3.47 ± 0.39		
	Rural	268 (46.2)	3.57 ± 0.43		
Native province	Yes	478 (82.4)	3.54 ± 0.41	–0.980	0.327
	No	102 (17.6)	3.52 ± 0.45		
Highest education	Polytechnic school	13 (2.2)	3.47 ± 0.45	15.054	0.002**
	Junior college	192 (33.1)	3.52 ± 0.44		
	Bachelor	365 (62.9)	3.54 ± 0.40		
	Master or above	10 (1.7)	3.97 ± 0.28		
Department	Medical	177 (30.5)	3.55 ± 0.42	4.920	0.670
	Surgical	138 (23.8)	3.56 ± 0.39		
	Gynecological	51 (8.8)	3.54 ± 0.45		
	Pediatrics	52 (9.0)	3.45 ± 0.42		
	ICU	38 (6.6)	3.54 ± 0.33		
	Operating room	29 (5.0)	3.58 ± 0.44		
	Emergency	40 (6.9)	3.59 ± 0.48		
	Others	55 (9.5)	3.49 ± 0.44		
Work more than 40 h a week	Yes	313 (54.0)	3.52 ± 0.40	–0.978	0.328
	No	267 (46.0)	3.56 ± 0.44		
Only child	Yes	226 (39.0)	3.51 ± 0.41	–1.249	0.212
	No	354 (61.0)	3.56 ± 0.42		
Marital status	Married	81 (14.0)	3.57 ± 0.37	–0.917	0.359
	Unmarried	499 (86.0)	3.53 ± 0.43		
Have a preceptor	Yes	214 (36.9)	3.63 ± 0.45	–4.000	< 0.001***
	No	366 (63.1)	3.49 ± 0.39		
Held class cadre	Yes	108 (18.6)	3.62 ± 0.45	–2.039	0.041*
	No	472 (81.4)	3.52 ± 0.41		
Have part-time job experiences	Yes	121 (20.9)	3.63 ± 0.45	–2.584	0.010*
	No	459 (79.1)	3.51 ± 0.41		

ATMD, Adolescence Time Management Disposition Scale.

*p < 0.05, **p < 0.01, ***p < 0.001.

### Time management disposition level of new nurses

As shown in Table 2, new nurses presented an upper-middle level of TMD of 3.54 ± 0.42. The average point of sense of time’s value was the highest at 3.82 ± 0.52, followed by the sense of time efficacy at 3.60 ± 0.46, time monitoring view was the lowest at 3.40 ± 0.43. The individual orientation score was lower than the social orientation score in sense of time’s value subscale (*p* < 0.001). With respect to the time monitoring view dimension, the lowest and highest scores were the time allocation and offered target, respectively (*p* < 0.001). Meanwhile, new nurses performed higher on time management behavior than on time management the sense of time efficacy (*p* < 0.001).

**TABLE 2:** Descriptive statistical measurements of the scales and ATMD subscale (*N* = 580).

ATMD	Total scores	Items mean score	*Z/χ* ^2^	*p*
				
	Mean ± SD			
Total	155.68 ± 18.41	3.54 ± 0.42	773.650	<0.001***
Sense of time’s value	38.20 ± 5.22	3.82 ± 0.52	–6.548	<0.001***
Individual orientation	18.89 ± 2.78	3.78 ± 0.56		
Social orientation	19.31 ± 2.64	3.86 ± 0.53		
Time monitoring view	81.47 ± 10.37	3.40 ± 0.43	758.140	<0.001***
Offered target	17.53 ± 2.26	3.51 ± 0.45		
Plans	17.50 ± 2.29	3.50 ± 0.46		
Priorities	17.30 ± 2.28	3.46 ± 0.46		
Feedback	16.49 ± 2.43	3.30 ± 0.49		
Time allocations	12.65 ± 2.13	3.16 ± 0.53		
Sense of time efficacy	36.01 ± 4.62	3.60 ± 0.46	–8.367	<0.001***
Time management efficacy	17.79 ± 2.24	3.56 ± 0.45		
Time management behavior efficacy	18.22 ± 2.52	3.64 ± 0.50		

ATMD, Adolescence Time Management Disposition Scale.

***p < 0.001.

### The correlation between new nurses’ job stressors, general self-efficacy, clinical communication ability, safety behavior, and time management disposition

The scores of NJSS, GSES, NCCCS, and SBS were 2.28 ± 0.36, 2.62 ± 0.51, 3.77 ± 0.28, and 3.54 ± 0.49, respectively. In addition, there was a significant negative correlation between the total score of NJSS and ATMD (*r* = –0.391, *p* < 0.001). On the contrary, the GSES, NCCCS, and SBS were positive significant associations with ATMD (*r* = 0.401, 0.441, and 0.336, *p* < 0.001; See Table 3).

**TABLE 3:** Correlation between NJSS, GSES, NCCCS, SBS, and ATMD (*N* = 580).

Variables	Subscales	Scores/M ± SD	*r*	*p* ***
NJSS		2.28 ± 0.36	–0.391	<0.001
	Nursing professional and work	2.48 ± 0.46	–0.356	<0.001
	Time allocation and workload	2.70 ± 0.43	–0.365	<0.001
	Work environment and equipment	2.14 ± 0.69	–0.193	<0.001
	Patient care	2.32 ± 0.34	–0.295	<0.001
	Management and interpersonal	1.78 ± 0.56	–0.293	<0.001
GSES		2.62 ± 0.51	0.401	<0.001
NCCCS		3.77 ± 0.28	0.441	<0.001
SBS		3.54 ± 0.49	0.336	<0.001

ATMD, Adolescence Time Management Disposition Scale; NJSS, Nurses Job Stressors Scale; GSES, General Self-Efficacy Scale; NCCCS, Nurse Clinical Communication Competence Scale; SBS, Safety Behavior Scale.

***p < 0.001.

### Stepwise regression analysis of new nurses’ time management disposition

Variables were represented by scales other than ATMD and all demographic characteristics as independent variables, the stepwise regression analysis was used to introduce them into the corresponding regression equations. Table 4 shows that the TMD of new nurses was predicted by three kinds of job stressors (time allocation and workload, management and interpersonal, nursing professional and work), clinical communication ability, safety behavior, and without a preceptor (*F* = 61.013, *p* < 0.001, *R*^2^ = 0.390, adjust *R*^2^ = 0.383). A total of 38.3% of the variance in the TMD was explained by the above variables. Three kinds of job stressors and without a preceptor had negative effects on the TMD of new nurses, and others had the opposite effect.

**TABLE 4:** Stepwise regression analysis results with ATMD score (*N* = 580).

Model	Unstandardized coefficient		Standardized coefficients	*t*	*p*
	B	Std. Error Beta			
Constant	1.958	0.230		8.515	< 0.001***
Clinical communication competence	0.459	0.049	0.311	9.301	< 0.001***
Safety behavior	0.240	0.028	0.283	8.560	< 0.001***
Job stressors					< 0.001***
Time allocation and workload	–0.191	0.042	–0.195	–4.494	< 0.001***
Management and interpersonal	–0.126	0.029	–0.167	–4.267	< 0.001***
Nursing professional and work	–0.090	0.045	–0.098	–1.981	0.048*
Have a preceptor					
No	–0.061	0.029	–0.071	–2.136	0.033*

ATMD, Adolescence Time Management Disposition Scale.

*p < 0.05, ***p < 0.001.

## Discussion

The result of TMD was at a moderate level of 3.54 ± 0.42 according to the division of scores (Lavander et al., 2016). We found that new nurses generally have an acceptable TMD, which demonstrated new nurses’ attitudes and values toward time. It may be that the new nurse at this time has just been working for 2–3 months. The head nurse may take more notice of the new nurses’ job division and they can get guidance from the senior nurses. The rational division of labor and concentration of nurses’ working hours will also benefit patients (Lavander et al., 2016). Therefore, paying attention to new nurses’ time management competence may be associated with better clinical outcomes for patients.

With regard to the dimensions of the ATMD, the scores were ranked followed by the sense of time’s value, sense of time efficiency and time monitoring view, which was the same as in the previous research (Jvsu, 2011). New nurses undertook many clinical nursing tasks in their work. The work intensity and tasks were heavy, and they were involved in handling various emergencies. They faced the situation of working shifts, long working hours, and caring for more patients. Efficient use of time can ensure that the clinical work was carried out in an orderly manner and completed all tasks on time (Unruh and Nooney, 2011; HemmatiMaslakpak et al., 2016). This may be why new nurses scored highest in sense of time’s value. Meanwhile, the score on time efficiency wasn’t very low in this study. Hospital managers generally proposed transition programs for new nurses when they enter clinical work (Lea and Cruickshank, 2015). After clinical training, the new nurses have already mastered almost clinical tasks and were confident that they can complete them within the prescribed time. However, with the transformation of the medical model, the clinical nursing work was arduous, and various clinical emergencies were frequent (Rajabi et al., 2018), which made new nurses lacking time management knowledge and training. The worst was the shortage of time monitoring ability for them. Additionally, the score of social orientation was higher than individual orientation in sense of time’s value, which may demonstrate that new nurses were more concerned about the meaning of time for personal survival and development. For the highest and the lowest scores, target and time allocation were prominent among the time monitoring views. The translational shock made new nurses more focused on completing tasks within the prescribed time rather than on patient safety and holistic care (Murray et al., 2019). The time management efficacy score was lower than the time management behavior efficacy, which indicated that new nurses have more confidence in time management.

New nurses with a preceptor when first entering the clinical environment performed a higher score on TMD. Furthermore, without a preceptor also was a negative factor on ATMD. Preceptorship has been proposed to help new nurses transition smoothly (Clipper and Cherry, 2015). The preceptors can assist, clinical support and feedback to the new nurses, allowing them to build their skills and knowledge while providing safe and quality care to patients (Mellor and Greenhill, 2014). New nurses have difficulty in prioritization and time management, which may be improved by preceptors. (Shaw et al., 2018) suggested that in clinical work, preceptors should work closely with new nurses who have difficulty with time management and cannot perform their assigned duties by directly guiding time management skills.

The experience as a class cadre and part-time job will also influence the TMD for new nurses, which was consistent with a previous study on college students (Nian and Liu, 2012). The reason may be that new nurses who have served as class cadres and have done part-time jobs can accumulate more practical experience. In coordinating learning, working time, and completing work tasks, their time management disposition has been improved and developed to a certain extent. New nurses who have served as class cadres are more inclined to utilize their time to understand the sense of time value. Therefore, nursing students should be encouraged to serve as class cadres and energetic participants in activities that have a dual role in enhancing a proper understanding of the value of time and improving time management disposition.

Furthermore, new nurses had a higher score when they obtained a higher degree of educational background. Ebrahimi et al. (2014) also shown that nurses’ time management disposition was significantly correlated with education level. The participants of this study were new nurses who had worked for only one year, they generally had not yet accumulated the ability to allocate their clinical time, nor had they been able to upgrade their academic qualifications. Therefore, the currently highest academic credentials of new nurses have significantly better time management disposition.

As for general self-efficacy, the score of GSES was positively correlated with the ATMA’s score, which was similar to the findings of previous studies (Jun, 2017). Xie et al. (2020) also indicated that self-efficacy is a predictor of time management disposition in nursing managers. In view of the significant effect of general self-efficacy on TMD, the authors tend to believe that an improvement in general self-efficacy is necessary to contribute to the value of time management and time effectiveness.

New nurses’ TMD was negatively correlated with job stressors and positive correlation with clinical communication skills, and safety behavior. Insufficient professional ability, time allocation and heavy workloads, prioritized patient care, and an inadequate support system were all sources of stress for new nurses (Hu et al., 2017; Labrague and McEnroe-Petitte, 2018). Time allocation and workloads were common stressors for new nurses in the above research. However, new nurses’ TMD was ignored to consider in the transition program in past studies (Lea and Cruickshank, 2015). New nurses with a higher level of TMD have lower work stress and a strong sense of competence. Safety behavior contributed to reducing the incidence of adverse events during the clinical environment. But time management and medical errors have been a source of stress in the first few months of new nurses’ transition to the role of registered nurses (Murray et al., 2020). During the first few months, we suggested planning and providing relevant information and education to assist reduce the risk of safety errors during the transition. Meanwhile, effective communication and patient safety were important for new nurses. Algoso et al. (2019) indicated that building confidence in clinical practice and nursing by mastering basic communication and time management disposition for nurses. And register nurses’ preceptors attached great importance to effective communication, time management disposition, prioritization, and caring or sympathy (Shaw et al., 2018).

### Limitations

There were a few limitations in this study. First, the data collection in this study was completed at a one-time point and based on self-report, which may adversely affect the accuracy of the results. Second, we did not have the control group to understand the further effectiveness of TMD in new nurses in this study. Third, this study only revealed the TMD of new nurses, and further research is needed on specific interventions to improve time management disposition.

## Conclusion

The situation of new nurses’ TMD in China was at a moderate level. New nurses perform best in terms of time value, followed by time efficacy and time monitoring perspective. The TMD of new nurses was positively related to communication skills, and safety behavior, while negatively related to job stressors and new nurses without preceptors. Furthermore, these related factors were the TMD’s dominant related factors. In general, with an increase in work years and the accumulation of more work experience, the corresponding time management disposition of new nurses may also improve.

## Relevance for clinical practice

Clinical nurse managers should provide effective time management disposition support for new nurses, reasonably arrange new nurses’ scheduling and work assignments to relieve the work stress. Provide learning opportunities in terms of time management disposition for new nurses. After the new nurse entered the clinical environment, a preceptor should guide the new nurse through the work and life difficulties and psychological troubles. Besides, when recruiting new nurses, it is necessary to appropriately raise the educational threshold, and new nurses who have experienced in part-time cadres can focus on consideration.

## Data availability statement

The raw data supporting the conclusions of this article will be made available by the authors, without undue reservation.

## Author contributions

JX, JY, and LL conceived this study. XW, JL, XL, and PX participated in sampling methods design. SW and ZZ participated in statistical methods design. SD and AC drafted the protocol. JY and LL were assigned to be the corresponding author. All authors collaboration with the manuscript, read, and approved the final manuscript.

## Footnotes

1
http://www.nhc.gov.cn/
